# Supplementary material for: Modeling glioblastoma heterogeneity as a dynamic network of cell states
Source: Mol Syst Biol. 2021 Sep 16;17(9):e10105. doi: 10.15252/msb.202010105 (PMC8444284; doi:10.15252/msb.202010105)
Supplement: Supplementary file 6 — Source Data for Figure 5 [file MSB-17-e10105-s004.zip › Figure5A_sourcedata/GSEA_3017/hallmarks_stateD.GseaPreranked.1621934542601/HALLMARK_IL2_STAT5_SIGNALING.html]

Details for gene set HALLMARK\_IL2\_STAT5\_SIGNALING[GSEA]

|  || Dataset | state23017 |
| Phenotype | NoPhenotypeAvailable |
| Upregulated in class | na\_neg |
| GeneSet | HALLMARK\_IL2\_STAT5\_SIGNALING |
| Enrichment Score (ES) | -0.33959815 |
| Normalized Enrichment Score (NES) | -1.3383086 |
| Nominal p-value | 0.14806111 |
| FDR q-value | 0.26824933 |
| FWER p-Value | 0.989 |
Table: GSEA Results Summary

  

Fig 1: Enrichment plot: HALLMARK\_IL2\_STAT5\_SIGNALING      
 Profile of the Running ES Score & Positions of GeneSet Members on the Rank Ordered List

  

| PROBE | GENE SYMBOL | GENE\_TITLE | RANK IN GENE LIST | RANK METRIC SCORE | RUNNING ES | CORE ENRICHMENT || 1 | PHLDA1 |  |  | 59 | 0.336 | -0.0096 | No |
| 2 | MYC |  |  | 77 | 0.313 | 0.0108 | No |
| 3 | POU2F1 |  |  | 194 | -0.253 | -0.0516 | No |
| 4 | ITGAV |  |  | 217 | -0.255 | -0.0413 | No |
| 5 | IFNGR1 |  |  | 288 | -0.263 | -0.0672 | No |
| 6 | ANXA4 |  |  | 333 | -0.267 | -0.0727 | No |
| 7 | PRNP |  |  | 344 | -0.267 | -0.0518 | No |
| 8 | PLEC |  |  | 355 | -0.268 | -0.0309 | No |
| 9 | MAP6 |  |  | 369 | -0.270 | -0.0121 | No |
| 10 | GLIPR2 |  |  | 411 | -0.275 | -0.0144 | No |
| 11 | WLS |  |  | 456 | -0.280 | -0.0184 | No |
| 12 | CASP3 |  |  | 459 | -0.280 | 0.0100 | No |
| 13 | CSF1 |  |  | 614 | -0.303 | -0.0763 | No |
| 14 | CCNE1 |  |  | 850 | -0.357 | -0.2193 | No |
| 15 | RRAGD |  |  | 928 | -0.386 | -0.2374 | No |
| 16 | TNFRSF21 |  |  | 964 | -0.399 | -0.2218 | No |
| 17 | CDC6 |  |  | 1008 | -0.418 | -0.2103 | No |
| 18 | SERPINB6 |  |  | 1057 | -0.449 | -0.1994 | No |
| 19 | HOPX |  |  | 1240 | -0.672 | -0.2680 | Yes |
| 20 | APLP1 |  |  | 1249 | -0.700 | -0.1995 | Yes |
| 21 | CD44 |  |  | 1270 | -0.762 | -0.1336 | Yes |
| 22 | SPP1 |  |  | 1277 | -0.787 | -0.0543 | Yes |
| 23 | IFITM3 |  |  | 1279 | -0.812 | 0.0316 | Yes |
Table: GSEA details [plain text format]

  

Fig 2: HALLMARK\_IL2\_STAT5\_SIGNALING: Random ES distribution      
 Gene set null distribution of ES for **HALLMARK\_IL2\_STAT5\_SIGNALING**
